# European ILD registry algorithm for self-assessment in interstitial lung diseases (eurILDreg ASA-ILD)

**DOI:** 10.1371/journal.pone.0316484

**Published:** 2025-01-29

**Authors:** Ekaterina Krauss, Laurenz H. Claas, Silke Tello, Jennifer Naumann, Sandra Wobisch, Stefan Kuhn, Raphael W. Majeed, Karen Moor, Maria Molina-Molina, Oisin Byrne, Rebecca Borton, Marlies S. Wijsenbeek, Nik Hirani, Carlo Vancheri, Bruno Crestani, Andreas Guenther, on behalf of eurILDreg investigators

**Affiliations:** 1 European IPF/ILD Registry and Biobank (eurIPFreg/bank, eurILDreg/bank), Giessen, Germany; 2 Center for Interstitial and Rare Lung Diseases, Universities of Giessen and Marburg Lung Center (UGMLC), Justus-Liebig-University Giessen, Member of the German Center for Lung Research (DZL), Germany; 3 Cardio-Pulmonary Institute (CPI) Klinikstr, Giessen, Germany; 4 Centre of Excellence for Interstitial Lung Diseases and Sarcoidosis, Department of Respiratory Medicine, Erasmus University Medical Centre, Rotterdam, The Netherlands; 5 ILD Unit, Respiratory Department, Biomedical Research Institute of Bellvitge (IDIBELL), University Hospital of Bellvitge-IDIBELL, Barcelona, Spain; 6 patientMpower, The Digital Hub, Dublin, Ireland; 7 Centre for Inflammation Research, University of Edinburgh, Edinburgh, United Kingdom; 8 Regional Referral Center for Rare Lung Diseases, University Hospital Policlinico, Department of Clinical and Experimental Medicine, University of Catania, Catania, Italy; 9 Institute National de la Sainté et de la Recherche Médicale, Hopital Bichat, Service de Pneumologie, Paris, France; 10 Agaplesion Lung Clinic, “Evangelisches Krankenhaus Mittelhessen”, Giessen, Germany; University of Turin: Universita degli Studi di Torino, ITALY

## Abstract

**Background and aims:**

Predicting progression and prognosis in Interstitial Lung Diseases (ILD), especially Idiopathic Pulmonary Fibrosis (IPF) and Progressive Pulmonary Fibrosis (PPF), remains a challenge. Integrating patient-centered measurements is essential for earlier and safer detection of disease progression. Home monitoring through e-health technologies, such as spirometry and oximetry connected to smartphone applications, holds promise for early detection of ILD progression or acute exacerbations, enabling timely therapeutic interventions.

**Methods:**

The European ILD Registry Algorithm for Self-Assessment in ILD (eurILDreg ASA-ILD), developed by all eurILDreg principal investigators, includes questionnaires on symptom burden, respiratory infections, and quality of life (EQ5D VAS, K-BILD, LCQ). The algorithm also incorporates spirometry and oxygen saturation measurements, both at rest and during exercise (one-minute sit-to-stand test, 1STST). This ASA-ILD algorithm is integrated into the patientMpower Ltd. smartphone application, used for patient-led monitoring, research, and clinical care since 2016, and available on both Apple and Android platforms.

**Discussion:**

For patient-centered measurements, participants in the multicenter eurILDreg study will receive a patientMpower account, a handheld clinical-grade spirometer (Spirobank Smart, MIR, Italy), and a pulse oximeter (Nonin Medical, Inc. Plymouth, MN, USA), along with usage instructions. Artificial intelligence software (ArtiQ) will analyze spirometry maneuvers in real-time, ensuring compliance with recent ERS/ATS criteria and providing automated feedback. Pulse oximetry is integrated into the exercise testing within the application, following an automated in-app protocol developed with clinician involvement for safety and accuracy. The application will send reminders to participants to complete patient-reported outcome measures (PROMs) according to the study protocol.

**Conclusion:**

This study is designed to explore the potential of e-Health technologies, such as home monitoring via spirometry and oximetry, integrated with the eurILDreg ASA-ILD algorithm and patientMpower app, to improve early detection and management of ILD. A pilot trial showed promising adherence to spirometry, indicating that digital health interventions could enhance patient care and outcomes in ILD.

**Trial registration:**

The ethics committee of the Justus-Liebig-University of Giessen has approved the eurILDreg and this substudy with the protocol reference number 111/08. The research was conducted strictly according to the principles of the Declaration of Helsinki. Patients were included into the registry upon having signed the informed consent. The eurIPFreg and eurIPFbank are listed in ClinicalTrials.gov (NCT02951416). EurILDreg is registered in German Clinical Trials Register, DRKS 00028968.

## Background

Interstitial lung diseases (ILDs) are a diverse group of chronic lung diseases affecting the respiratory unit of the lung, which are historically classified in four groups: ILDs with a known cause, idiopathic interstitial pneumonias (IIPs), granulomatous disorders, as well as rare ILDs [1]. Course and prognosis of ILD varies significantly between different entities [2]. Some ILDs are reversible, other ILDs have the potential for stabilization, but fibrotic ILDs are often progressive (progressive pulmonary fibrosis, PPF) and ultimately fatal, especially idiopathic pulmonary fibrosis (IPF) [3,4]. Therefore, pharmacological and non-pharmacological treatment strategies differ between ILDs and even within the same diagnosis. Likewise, care needs may differ within ILD entities [5]. Currently, there are no routinely used biomarkers which can accurately predict disease course for individual patients [6,7].

The concept of patient-centered ILD care is well-recognized but remains challenging. While patients and healthcare providers understand the importance of focusing on patient needs, preferences, and collaboration, implementing these practices in real-world settings is difficult. This difficulty is due to limited time and resources available to healthcare professionals, which can hinder their ability to fully engage in patient-centered care practices [8].

E-Health technologies, exemplified by innovations such as home monitoring, have the capacity to foster patient-centered care and enhance healthcare quality through the utilization of information communication technology [9]. This allows remote exchange of data between patients and healthcare professionals and enables monitoring, research and management of long term conditions [10]. Frequent home monitoring, utilizing physiological measures, and Patient-Reported Outcome Measures (PROMs), has the potential to enable early detection of disease progression and may allow timely intervention, especially in IPF and PPF. Spirometers and oximeters connected to smartphone applications provide a suitable solution for physiological assessment [11].

In this regard, home monitoring has gained increasing attention in the last years [12,13]. Few studies using daily handheld spirometry have been performed in ILD patients [14]. These studies showed that home-based measurement of pulmonary function using a handheld spirometer is, in principle, feasible, reliable and has an acceptable variability [15]. Moreover, in some studies home spirometry has shown to be more sensitive in predicting disease progression and mortality than in-hospital spirometry [16,17]. However, data from randomized clinical trials have shown that self-measurements must be assessed for quality and filtered for inconsistent and implausible data. Overall, home spirometry presents an attractive home monitoring approach suitable for both clinical practice and research.

Another commonly used physiological measure in ILDs is the 6-minute walking test (6MWT), which evaluates exercise capacity and exercise-induced hypoxemia [18]. This test is frequently performed in hospitals, however, it would be challenging to perform a 6MWT at home due to the lack of standardized environment for the testing (i.e., availability of a 30-m corridor) [19]. In recent years, the 1 minute sit to stand test (1STST) has been proposed as a reliable, safe alternative to evaluate exercise capacity in patients with ILD; the 1STST is much easier to perform at home, as only a chair is needed [20].

## Methods

### Objectives

The primary objective of this project is to develop an algorithm for self-assessment (ASA) of symptom burden, respiratory infection surrogates, and quality of life (QoL: EQ5D VAS, K-BILD, LCQ), and to implement this algorithm in the patientMpower (pMp) app. Additionally, we aim to include spirometry tests and measurements of oxygen saturation at rest and during exercise (using a 1STST) into the ASA-ILD.

### Inclusion and exclusion criteria

Patients with ILD have been recruited from ILD centers in Giessen (Germany), Barcelona (Spain), and Catania (Italy) since April 2023. Recruitment at other European registry for Interstitial Lung Diseases (eurILDreg) sites is pending ethics approval and is expected to begin soon. Inclusion criteria require patients to be 18 years or older, diagnosed with ILD according to current guidelines, and confirmed through local multidisciplinary discussion (MDD) boards. Exclusion criteria include inability to comply with smartphone and app requirements, as determined by the investigator and/or patient, lack of internet access, and no intention to participate in measurements within the subsequent study year. The study also includes drop-out criteria such as low compliance, loss or misuse of medical devices, and progression to clinical conditions requiring palliative care. In cases of drop-out, data collected up until the point of withdrawal will be included in the final analysis, provided it meets predefined quality and completeness standards.

### Ethics approval and consent to participate

The ASA-ILD algorithm is a substudy of the eurILDreg, which has been reviewed and approved by the local ethics committee (AZ 111/08, Amendment dated March 20, 2013) [21,22]. All participating sites of eurILDreg must obtain approval from their respective Medical Ethics Committees. The study will be conducted in accordance with the Declaration of Helsinki. Written informed consent will be obtained from the participants. The EurILDreg is registered in the German Clinical Trials Register under DRKS 00028968 [23].

### Study endpoints

Primary endpoint:

- Serial lung function measurement (including forced vital capacity, FVC) and oxygen saturation measurements throughout the one-year study period.

Secondary endpoints:

- Comparison of serial lung function measurements obtained from home and hospital spirometry, focusing on: [1] comparing the reproducibility of spirometry between home and hospital settings, and [2] evaluating the accuracy of home spirometry in identifying reliable progression trajectories of lung function over time.
- Assessment of participant adherence to scheduled measurements.
- Exploration of correlations between pulmonary function tests and PROMs at baseline, 3 months, 6 months, and 12 months.

Exploratory endpoints:

- Examination of participant adherence to spirometry and 1STST
- Monitoring of changes in Health-Related Quality of Life (HRQoL) and symptom burden
- Analysis of hospitalizations rates, disease progression, acute exacerbations, together with overall outcomes
- Evaluation of healthcare provider and participant satisfaction, along with their experiences with the pMp application
- Assessment of adherence to the home monitoring by evaluating data set completeness, including both pulmonary function and PROMs.

### Study design

PMp applications have been specifically developed for ILD and have been used in patient-led monitoring, research, and clinical care pathways since 2016. The app is available for both Apple and Android operating systems.

The eurILDreg ASA-ILD works with clinical-grade medical devices, including a spirometer (Spirobank Smart, MIR, Italy), and a pulse oximeter (Nonin Medical, Inc., Plymouth, MN, USA), both of which can be seamlessly connected to the mobile pMp application via Bluetooth. At the time of inclusion, participants will be provided with a pMp account, a handheld spirometer, and a pulse oximeter, along with detailed instructions. Via the pMp application, online home spirometry, measurements of oxygen saturation at rest and during a 1STST, completion of PROMs, and a daily symptom check are conducted. The app shows data from the nearest weather station indicating outdoor pollution level.

**Fig 1:**
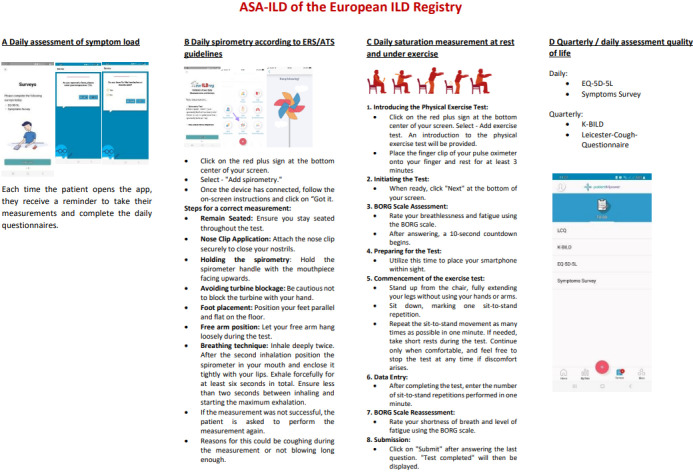
ASA-ILD measurement algorithm. Abbreviations: ASA-ILD, algorithm for self-assessment in interstitial lung diseases.

Patients will receive comprehensive guidance on study procedures, including instructions for conducting lung function and exercise tests (Fig 1). They will then download the application in their native language, input login credentials, and establish Bluetooth connections with devices to ensure accurate measurements. Study participants will be asked to conduct daily lung function assessments and monitor oxygen levels both at rest and during exercise each morning, around the same time to reduce variability. Before these assessments, patients share their daily state by responding to a brief set of questions (Symptoms-Check). Engaging with the measurements requires approximately 3–5 minutes of the patient’s time each day.

Spirometry maneuvers performed using the application are analyzed in real time with artificial intelligence software (ArtiQ) for compliance with recent ERS/ATS criteria [24,25]. Pulse oximetry is used for exercise testing within the app, following an automated protocol developed with clinician involvement, ensuring safety and accuracy. The app also sends reminders to participants to complete PROMs at the frequency specified in the study protocol.

The finalized ASA-ILD protocol ensures that eurILDreg patients who meet study eligibility criteria will receive study information through their ILD treatment centers. Patient instructions for using the spirometer include inserting batteries, ensuring it remains in perpetual pairing-ready mode, changing batteries every 4–6 months, cleaning the mouthpiece and rotor weekly, and demonstrating proper device handling. Participants using the Nonin Pulse Oximeter are advised to replace batteries every 6 months or as needed for optimal performance, and to maintain a clean, dry finger for accurate readings. Additionally, participants are requested to complete the EQ5, KBILD, and LCQ questionnaires every three months, providing valuable ongoing research data.

Following a demonstration conducted by trained personnel at the ILD center, patients proceed by tapping the red ‘plus’ sign on the home screen of their smartphones, selecting ‘add spirometry’ and allowing a moment for the app to establish a connection to the spirometer. Once connected, they select ‘understood’ to initiate the test and to do the spirometry measurement (blow through visual and audible cues). They then receive feedback on the test, including any substandard techniques, and are asked to repeat the measurement. Any improper exhalation detected prompts an explanatory error message and asks to repeat the measurement. The spirometry measurements adhere to the recent ERS/ATS guidelines in regard to technical standards on interpretive strategies for lung function tests [24,25].

For the 1STST, patients need a chair with a standard height of around 46 centimeters, without armrests, positioned against a wall. Patients are instructed to sit upright with their knees and hips bent at 90-degree angles, feet placed flat on the floor hip-width apart, and arms resting firmly on their hips. Prior to the test, the patient undergoes a pre-test check of their oxygen saturation to ensure it is above 94%. Additionally, the test will alert the patient and stop if they begin desaturating during the test. During the 1-minute test, patients perform repetitions of standing up and sitting down in this specific position at a pace comfortable for them, without using their arms for support. They aim to complete as many repetitions as possible within the allotted time. If needed, patients are allowed to take short rests during the 1-minute period. In the application, patients document the number of repetitions they achieve, as well as report their level of breathlessness (Borg Dyspnea score). Oxygen saturation levels are recorded automatically. This process ensures accurate and comprehensive data collection for the study.

Patients assess their data by selecting the “my data” option at the bottom of the home screen and choosing a timeframe from the options at the top of the screen. Patients are encouraged to contact the clinic for any assistance needed with device use. Automated alerts are not generated for healthcare providers in the event of alterations in pulmonary function, oxygen saturation or PROMs. In general, during the study period, patients are not called up or re-contacted outside their visits to the center.

Prior to initiating the exertion test, patients will undergo a pre-test oxygen saturation assessment. If their oxygen saturation is below 94%, the test will not proceed to mitigate the risk of desaturation. Similarly, if a patient’s oxygen saturation drops below 94% during the test, it will be immediately discontinued to prevent further desaturation.

Although, the study team will not actively monitor the real-time data collected through the application; however, patients will have access to their data and will receive clear guidance on how to interpret it. While the app itself will not prompt any actions based on the readings, patients will be instructed on the appropriate steps to take should they observe concerning trends. For example, if patients notice a significant decline in their lung function readings, they will be advised to contact their physician promptly. This approach empowers patients to take an active role in managing their health while ensuring they know when to seek professional assistance.

The study protocol includes strategies for addressing data transfer issues. Patients will see an error screen with a description of the connection issue they are experiencing, in case of failed or incomplete data transfer. Additionally, a support line will be available to guide patients in resolving these issues.

While the real-time data will not directly influence the primary and secondary outcomes during the study, it provides valuable insights into patient trends and health status. These data will be analyzed post-study to identify patterns and correlations that may contribute to understanding the effectiveness of the remote monitoring approach. Additionally, the study design will include a framework for assessing how variations in real-time data correlate with changes in primary and secondary outcomes, enabling us to explore the potential impact of real-time monitoring on patient care and health outcomes in the future. This comprehensive analysis will help determine whether integrating real-time data into clinical decision-making enhances patient management and improves health outcomes in patients with ILD.

### Data transfer and protection

PMp, a private company registered in Ireland under number 563516 and headquartered at The Digital Hub, Dublin 8 Ireland D08TCV, is dedicated to handling personal data in compliance with its Data Processing Agreement. Both pMp and eurILDReg have committed to adhering to all relevant laws and regulations governing the storage, use, and disclosure of protected health information. This includes Regulation (EU) 2016/679 (General Data Protection Regulation), which was adopted by the European Parliament and the Council on April 27, 2016, concerning the protection of individuals regarding the processing of personal data and the free movement of such data, and the repeal of Directive 95/46. Additionally, they have pledged to comply with the Irish Data Protection Acts 1988 to 2018 and any current or future regulations issued under either the GDPR or the Irish Data Protection Acts 1988 to 2018.

Assessment results and measurements are transmitted as real-time data to the pMp database on a monthly basis, utilizing a secure, encrypted connection and stored on a secure server. All data are kept strictly confidential, and the app does not collect any personally identifiable information. Periodically, data from the pMp database are transferred to the eurILDreg Data Warehouse (DWH) through a secure, encrypted connection.

### Strategies to ensure patients’ compliance

Clinical studies assessing the feasibility and patient acceptability of remote monitoring for ILD using the pMp platform have included a wide age range of participants, including those over 75 years. Notably, Edwards et al. reported a mean participant age of 68 years with a standard deviation of ± 11 years [26]. Given that advancing age is often associated with declining visual acuity, the application has been optimized to support larger font sizes for improved readability on mobile devices.

For participants concerned about technical proficiency, in-person or remote technical support will be available for troubleshooting. The interface is designed for simplified, intuitive navigation to accommodate varying levels of technical proficiency. The pMp platform aims to achieve 99.99% availability, excluding any pre-scheduled maintenance periods. A service level agreement is established with the application provider, detailing response times and target resolution timelines based on the severity of support requests. Technical support is offered directly to patients in both English and Spanish via chat, phone, or email, while communication in other languages is coordinated through local clinicians at participating sites. The app features an intuitive user interface and clear, step-by-step instructions to facilitate navigation.

The pMp app will provide automated reminders for key tasks, such as lung function tests and symptom reporting, to keep participants engaged. To ensure patient compliance, a comprehensive strategy will be implemented that includes regular follow-ups, and targeted interventions to address common barriers such as age-related challenges, technical literacy, and device fatigue. Participants will attend scheduled follow-up visits at ILD centers every 3 to 6 months, allowing the study team to assess adherence, troubleshoot issues, and provide additional guidance and motivation.

To mitigate device fatigue, the app will limit the frequency of required inputs, reducing participant burden while still capturing essential data. This strategy aims to maintain engagement and prevent participants from feeling overwhelmed by frequent interactions. Non-compliant patients, defined as those who have not taken any measurements for four consecutive weeks, will be categorized into a separate study subgroup. Sensitivity analyses will assess the impact of non-compliance on primary and secondary outcomes, allowing differentiation between compliant and non-compliant participants. This will also facilitate the exploration of potential reasons for non-compliance, such as age, disease severity, or technical difficulties.

By integrating regular follow-up mechanisms, tailored support for diverse patient needs, and a clear plan for managing non-compliance, this strategy aims to enhance overall adherence, minimize data loss, and ensure the reliability of the study’s findings.

### Risk mitigation strategies for technical failures

When conducting a clinical study utilizing remote monitoring technology, such as the pMp platform, it is essential to implement robust risk mitigation strategies to address potential technical failures. To facilitate this, technical support will be accessible to patients through various channels, including phone, chat, and email. A dedicated support team will be specifically trained to assist with issues related to the app and connected devices.

Before the study commences, participants will undergo comprehensive training sessions covering app usage and troubleshooting common issues. Those experiencing difficulties with their devices will receive guidance through troubleshooting steps. Should a device be identified as faulty, a replacement will be sent promptly to the participant. To ensure timely replacements, a sufficient inventory of devices will be maintained, which will also involve tracking device status and the frequency of replacements to identify any recurring issues with specific models or batches.

Additionally, a disaster recovery plan will be established to manage large-scale technical disruptions, such as app outages or data syncing issues. This plan will outline predefined response protocols to swiftly restore functionality and ensure the security of patient data. Regular maintenance and updates of the app will be scheduled during low-usage periods to minimize disruption and enhance system reliability, with participants notified in advance of any planned downtime for maintenance activities.

By proactively addressing these areas, the study can effectively mitigate risks associated with technical failures, ensuring participants receive the necessary support and that data integrity is maintained throughout the research process. This comprehensive approach will enhance the reliability of the study’s findings while simultaneously improving participant satisfaction and adherence.

### Results of a pilot trial

The study commenced with an evaluation of adherence and feasibility through a pilot trial involving 25 participants over a 12-week period. During this phase, adherence to the algorithm was assessed by tracking the number of completed spirometry and 1STST measurements per day or week throughout the study period. Our data demonstrated considerable variability in adherence levels to spirometry and 1STST measurements.

**Fig 2:**
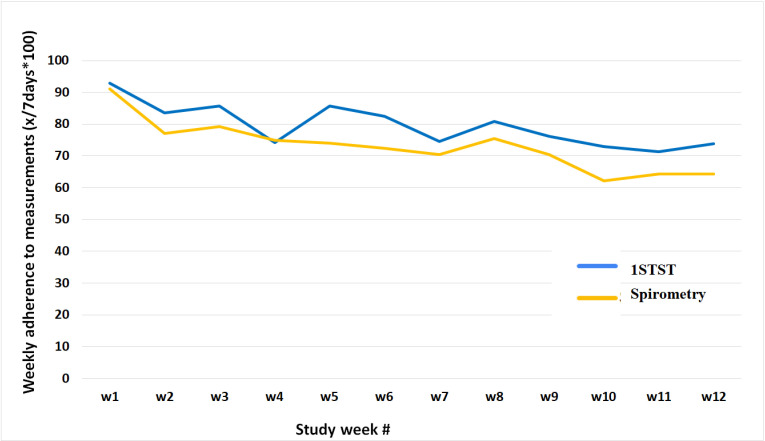
Adherence rates for spirometry and the one-minute sit-to-stand test in the adherent cohort (pilot study). Abbreviations: 1STST, one-minute sit-to-stand test; w, week.

**Fig 3:**
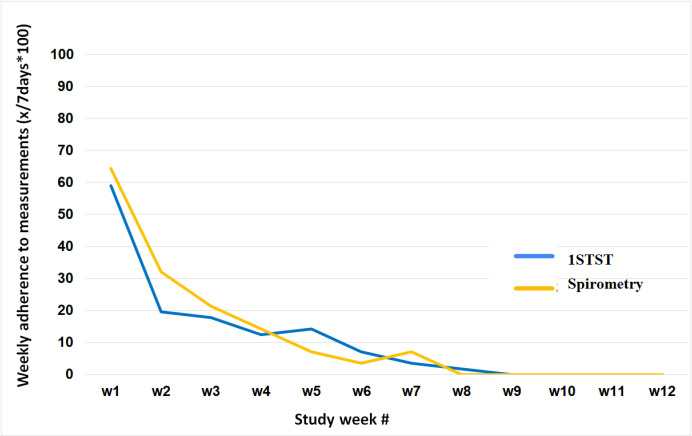
Adherence rates for spirometry and the one-minute sit-to-stand test in the non-adherent cohort (pilot study). Abbreviations: 1STST, one-minute sit-to-stand test; w, week.

The mean adherence rate for spirometry measurements was found to be 91%, and no significant limitations in device use were identified. The individual weekly adherence varied significantly, with some patients’ consistently achieving 100% adherence while others lost interest early on. When defining adherence as at least one spirometry or 1STST test per week, we found 84% adherent and 16% non-adherent for spirometry (Fig 2), and 64% adherent and 36% non-adherent for 1STST (Fig 3) over an observation period of 12 weeks. Mean adherence rates over time remained stable in adherent patients over 12 weeks, whereas non-adherent patients showed a gradual decline to 0% adherence by week 8. Notably, adherence rates did not significantly differ between spirometry and 1STST groups.

The main study is currently ongoing with continuous patient recruitment; to date, a total of 62 patients have been recruited.

## Discussion

Registry data is especially valuable for rare heterogeneous diseases, providing real-life insights into disease behavior and treatment response. Unlike randomized controlled trials, registries operate with less strict inclusion criteria, making them more aligned with the realities of daily clinical practice [23]. Anticipated to gain valuable insights, this prospective ongoing study focuses on online home monitoring in ILD patients. The notable adherence observed in the initial pilot trial indicates a general willingness and capability among patients to actively participate in spirometry and exercise tests. This positive response suggests that digital tools, such as the pMp application and handheld devices, might be integrated into the self-monitoring routines of ILD patients. However, there might be some variability in adherence levels, emphasizing the diverse nature of patient behavior and underscoring the importance of adopting a tailored approach when implementing the digital tools.

Prior research has highlighted the potential benefits of online home monitoring for ILD patients, demonstrating its advantages for both daily care and research. Althobiani et al analyzed thirteen studies with a total of 968 patients, with nine of the studies indicating that mean adherence to home monitoring exceeded 75%, and a strong correlation (ranging from 0.72 to 0.98, p < 0.001) was found between home and hospital-based readings [9,27].

Additionally, two studies suggested that home monitoring of FVC could aid in detecting progression in IPF, ultimately highlighting the feasibility and potential benefits for patients with ILD [27]. Moor et al showed that home monitoring in ILD showed a tendency to enhance psychological well-being, and it was highly valued by patients, enabling personalized medication adjustments [11]. Johannson et al showed the home monitoring improved endpoint efficiency in IPF, the mean adherence was 90.5% (SD = 18.3%) over 24 weeks [28]. Veit et al showed that median adherence dropped within the first 28 days and decreased from 90% to 81% over 6 months; therefore, daily home spirometry seem to be feasible in ILD and facilitates the identification of FVC variability, which is associated with disease progression [29]. Recently, Nakshbandi et al. introduced an international e-Health ILD home monitoring study, in which 700 patients aspire to engage in a home monitoring program for a duration of two years [30].

The limitations of a one-size-fits-all strategy become evident in the last years, urging healthcare providers and researchers to prioritize individual patient preferences, motivations, and potential barriers in the implementation of digital health solutions. The implications drawn from study design are expected to offer a glimpse into the promising future of digital health tools, showcasing their potential to revolutionize patient engagement and self-monitoring in the ILD management with a more personalized approach.

Looking ahead, there is an urging need for sustained research and development in the field of digital health for ILD. This study design underscores the need of continuous exploration, required to refine and advance the e-Health technologies, ensuring their effectiveness and adaptability in real-world settings. The emphasis on ongoing patient education, support, and regular follow-ups emerges as a crucial aspect for maintaining engagement and unlocking the full potential of technological interventions. By doing so, we envisage an optimized landscape for the monitoring and care of ILD patients, foreseeing the potential for improved outcomes and an enhanced quality of life.

Understanding patient motivations, preferences, and potential challenges, our study sets the stage for future research endeavors. The imperative for this investigation becomes apparent, aiming for a comprehensive understanding of adherence dynamics. This involves delving into factors such as individual motivation, app complexity, health status, and the impact of external support.

As we move forward with this international collaboration between ILD research centers, healthcare providers and patients, unraveling these intricacies will contribute to the development of more effective interventions and inform strategies to interpret and improve adherence rates in the realm of digital health for ILD.

## Abbreviations

1STST: One-minute Sit-to-Stand Test
6MWT: Six-minute Walking Test
ASA-ILD: Algorithm for Self-Assessment in Interstitial Lung Diseases
ArtiQ: Artificial intelligence software used for real-time analysis of spirometry maneuvers
DRKS: German Clinical Trials Register
EQ5D VAS: EuroQol 5-Dimension Visual Analog Scale
ERS/ATS: European Respiratory Society/American Thoracic Society
eurILDreg: European Registry and Biobank for Interstitial Lung Diseases
Fig: Figure
FVC: Forced Vital Capacity
GDPR: General Data Protection Regulation
HRQoL: Health-Related Quality of Life
ILD: Interstitial Lung Diseases
IIPs: Idiopathic Interstitial Pneumonias
IPF: Idiopathic Pulmonary Fibrosis
K-BILD: King’s Brief Interstitial Lung Disease Questionnaire
LCQ: Leicester Cough Questionnaire
MDD: Multidisciplinary Discussion
MIR: Medical International Research (manufacturer of Spirobank Smart)
Nonin Medical, Inc: Manufacturer of the pulse oximeter used in the study
pMp, patientMpower Ltd.: Developer of the smartphone application
PPF: Progressive Pulmonary Fibrosis
PROMs: Patient-Reported Outcome Measures
QoL: Quality of Life
RCTs: Randomized Clinical Trials
